# Tuina therapy for temporomandibular joint disorder syndrome: A protocol for systematic review and meta-analysis

**DOI:** 10.1097/MD.0000000000024202

**Published:** 2021-01-22

**Authors:** Jing Chen, Yongchao Li, Lirong Zeng, Wei Sun, Ni Wei, Hui Xie, Wenjuan He

**Affiliations:** a School of Rehabilitation, Xiangnan University; b Chenzhou No.1 People's Hospital, Chenzhou, Hunan province, China.

**Keywords:** efficacy, protocol, systematic review, temporomandibular joint disorders, tuina

## Abstract

**Background:**

Temporomandibular joint disorders (TMD) is common in clinic at present, which seriously affects the mental health and quality of life of patients. With the development of society, the incidence of TMD is gradually increasing. At present, there are many treatment methods, Tuina as a characteristic traditional Chinese medicine therapy, clinical treatment of TMD has a significant effect. In recent years, there are many clinical studies on Tuina in the treatment of TMD, but the clinical efficacy of Tuina in the treatment of TMD has not been systematically evaluated. In this study, we systematically evaluated the relevant literature of Tuina in the treatment of TMD by using the method of evidence-based medicine, in order to provide reference for clinical research in this direction in the future.

**Methods:**

VIP Chinese database, China knowledge Network, Wanfang, China Biomedical Database, PubMed, Embase, Cochrane Library and Web of Science were searched for clinical randomized controlled trials of Tuina in the treatment of TMD from the establishment of the database to December 2020. The 2 researchers independently screened the literature and carried out quality assessment and data extraction for the included study, and used RevMan5.3 software for risk assessment and Meta analysis.

**Results:**

In this study, the efficacy and safety of Tuina in the treatment of TMD were evaluated by effective rate, visual analog score (VAS) of temporomandibular joint pain, dysfunction index ((DI), palpation index (PI), craniomandibular index (CMI), maximum mouth opening (MMO), incidence of adverse reactions and so on.

**Conclusion:**

This protocol can provide evidence-based basis for the treatment of TMD, with Tuina to significantly improve the symptoms and function of patients with TMD.

**OSF Registration number:**

DOI 10.17605/OSF.IO/J75A8.

## 1 Introduction

Temporomandibular joint disorder syndrome (TMD) is a group of diseases with clinical symptoms such as pain, bounce and limited opening of temporomandibular joint, involving temporomandibular joint or masticatory muscle system. It is a common disease in oral and maxillofacial region.^[1]^ With the development of society, various factors lead to a gradual increase in the incidence of TMD. It is reported that the prevalence rate of TMD is about 25%, seriously affecting the quality of life of patients.^[2]^ The pathogenesis of TMD includes mental, psychological, traumatic, occlusal, immune and other factors, but its cause is still not completely clear. Pain in patients with TMD is the primary symptom of patients seeking medical treatment.^[3–4]^ At present, there are many clinical treatment methods for TMD, and Tuina is one of the very important treatment methods.^[5–6]^

Tuina is a characteristic therapy of traditional Chinese medicine that uses hands or tools to treat patients’ body surface. It is widely used in clinic and has the effects of relieving pain, relieving muscle fatigue, promoting blood circulation and so on. Modern studies have shown that pressing the special position of patients with pain can have analgesic effect, and the analgesic effect is positively correlated with the content of β-endorphin in blood. Tuina can relieve pain by reducing the content of substance P (substance P), in blood and increasing the content of β-endorphin.^[7–8]^ At present, clinical studies show that Tuina has a significant effect in the treatment of TMD, which is worth popularizing widely.^[9]^ In recent years, there are many clinical studies on Tuina in the treatment of TMD, but there is no related systematic review. The purpose of this study is to objectively evaluate the efficacy and safety of Tuina in the treatment of TMD, and to provide reference for the clinical research and application of Tuina on TMD.

## 2 Methods

### 2.1 Protocol register

This protocol of systematic review and meta-analysis will be drafted under the guidance of the preferred reporting items for systematic reviews and meta-analysis protocols (PRISMA-P). It will be registered in the open science framework (OSF) on December 6, 2020 (registration number: DOI 10.17605/OSF.IO/J75A8).

### 2.2 Ethics

Since this is a protocol with no patient recruitment and personal information collection, the approval of the ethics committee is not required.

### 2.3 Eligibility criteria

#### 2.3.1 Types of studies

We will collect and search all the randomized controlled trials (RCT) of Tuina for TMD, regardless of the region of publication and blindness, but the search language is limited to Chinese and English.

#### 2.3.2 Object of study

Patients who can be clearly diagnosed as TMD, including gender, time of onset, age are not limited.

#### 2.3.3 Intervention measures

The treatment group was treated with simple Tuina therapy or Tuina combined with other therapy, while the control group was treated with other therapy except Tuina.

#### 2.3.4 Outcome indicators

1. Primary outcome: the wound healing rate;
2. Secondary outcomes: ①visual analog score (VAS); ②dysfunction index (DI); ③palpation index (PI); ④craniomandibular index (CMI); ⑤maximum mouth opening (MMO); ⑥incidence of adverse reactions.

#### 2.3.5 Exclusion criteria

1. only one article is selected for repeatedly published or repeatedly cited literature;
2. studies in which abstracts or full texts are not available;
3. studies in which the data are obviously incorrect or missing;
4. documents that do not indicate the author.

### 2.4 Retrieval strategy

The key words of “temporomandibular joint disorder syndrome”, “temporomandibular disorder”, “temporomandibular joint disorder”, “Tuina”, “Message” and “manipulation” were searched in Chinese database, while “temporomandibular joint disorders syndrome”, “TMD”, “Tuina”, “Message” and “manipulation” were searched in English database. Including China knowledge Network, Chongqing VIP Chinese Sci-tech Journal full-text Database, Wanfang, China Biomedical Database, PubMed, Embase, Cochrane Library, Web of Science, etc., clinical randomized controlled trials of Tuina in the treatment of TMD from the establishment of the database to December 2020. Take PubMed as an example, the retrieval strategy is shown in Table 1.

**Table 1:** Retrieval strategy in PubMed.

Number	Search terms
#1	Massage [Title/Abstract]
#2	Tuina [Title/Abstract]
#3	Manipulation [Title/Abstract]
#4	#1 OR #2 OR #3
#5	temporomandibular joint disorders [MeSH]
#6	TMD [Title/Abstract]
#7	temporomandibular disorders [Title/Abstract]
#8	#5OR #6 OR #7
#9	#4 AND #8

### 2.5 Data screening and extraction

**Figure 1:**
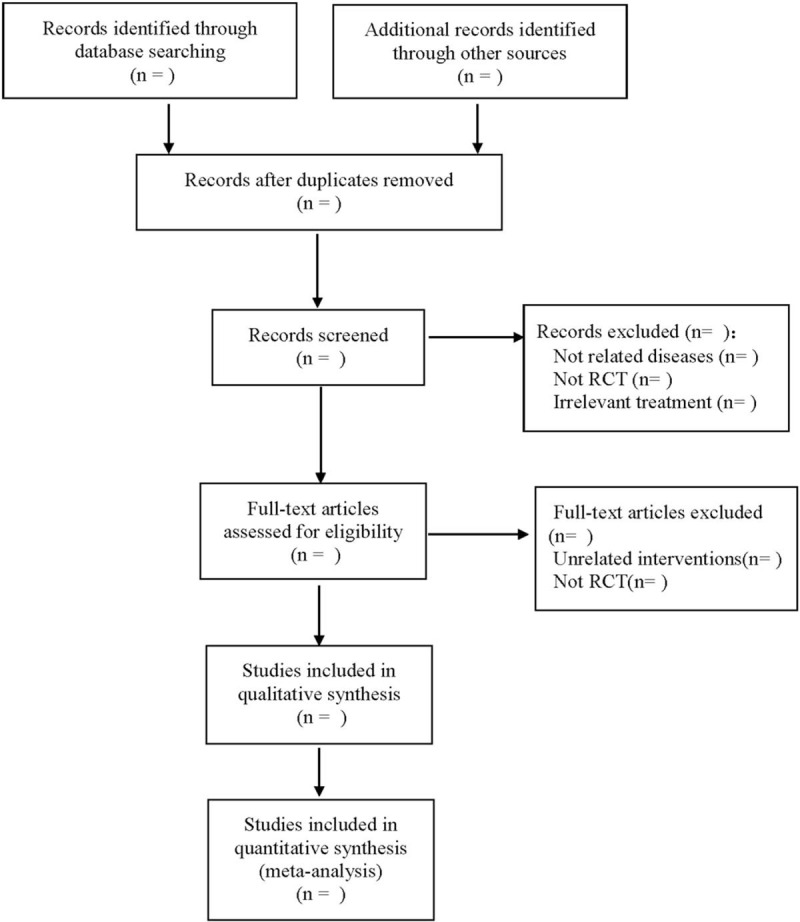
Flow diagram.

Referring to the Cochrane system evaluation manual, according to the flow chart of PRISMA, and according to the inclusion and exclusion criteria of the design scheme, the 2 researchers used EndNote X7 software to independently screen and extract data from the retrieval literature. If there is a dispute over the inclusion of the study, the 2 parties shall negotiate with each other or be decided by a third researcher. The 2 researchers used a data information extraction table designed in advance to extract the research content, including 1 basic data: title, publication date, journal, author, 2 research characteristics: number of cases, general demographic characteristics, intervention measures, follow-up, adverse events, etc. 3Indexes of outcome: effective rate, VAS, dysfunction index (DI), muscle tenderness index (PI), temporomandibular joint index (CMI), maximum mouth opening (MMO), incidence of adverse reactions. The screening process is shown in Figure 1.

### 2.6 Document quality evaluation

The bias risk assessment tool in Cochrane5.1.0 is used to evaluate the methodological quality of the included trials. The corresponding risk degree is judged by the 2 researchers from the aspects of Randomsequencegeneration, Allocationconcealment, Blindingofparticipantsandpersonnel, Blindingofoutcomeassessment, Incompleteoutcome Data, Selectivereporting and Otherbias, and the assessment results are cross-checked. If disputes are discussed with each other, if no agreement can be reached, it will be agreed with the third researcher. Finally, RevMan5.3 software is used to evaluate the bias risk of the study.

### 2.7 Statistical analysis

RevMan5.3 software was used for statistical analysis. The measurement data are expressed by mean difference (MD). When the units or measurement methods of the same effect index are not unified, they are expressed by standardized mean difference (SMD). The counting data use relative risk ratio (RR) as the statistical quantity of curative effect, and the 95% confidence interval (95%CI) is calculated. Heterogeneity test: Chi-Squared test was used, and the test statistic was *I*^2^. When there is statistical homogeneity among the studies (*P* > .1, *I*^2^ ≤ 50%), the fixed effect model is used for meta-analysis. When there is statistical heterogeneity among the studies (*P* ≤ .1, *I*^2^ > 50%), the source of heterogeneity needs to be analyzed. After excluding clinical and methodological heterogeneity, random effect model is used for Meta analysis. Subgroup analysis was used to deal with clinical heterogeneity, and if subgroup analysis could not be carried out, only descriptive analysis was carried out.

#### 2.7.1 Dealing with missing data

If there is a lack of data in the study, contact the author through the mailbox to obtain the relevant data. If there is no contact with the researcher or the research data has been lost, only descriptive analysis will be carried out.

#### 2.7.2 Subgroup analysis

According to the treatment group simple Tuina treatment of TMD and Tuina combined with other treatments for subgroup analysis; according to the course of treatment for subgroup analysis.

#### 2.7.3 Sensitivity analysis

In order to ensure the stability of the outcome index results, the sensitivity analysis of each outcome index was carried out.

#### 2.7.4 Assessment of reporting biases

If more than 10 articles were included in the outcome index literature, the funnel chart was used to evaluate the publication bias. In addition, Egger and Begg test were used for the evaluation of potential publication bias.

## 3 Discussion

According to the traditional theory of traditional Chinese medicine and the guidance of modern anatomy and physiology, Tuina applies various techniques to patients meridians and diseased parts to achieve the purpose of treating diseases. Relevant clinical studies have shown that Tuina manipulation can promote blood circulation, reduce blood pressure by increasing the activity of sodium pump in the blood, and Tuina can also regulate the immune function of the human body as a whole through neuro-endocrine-immunity; Tuina can repair injured skeletal muscle cells.^[10–12]^ It is reported that Tuina can reduce inflammatory factors such as tumor necrosis factor α and interleukin-6, increase the content of dopamine and β-endorphin, and relieve fatigue, inflammation and pain by eliminating lactic acid and blood urea nitrogen in the blood.^[13–15]^

The main symptoms of TMD include pain, joint bouncing and limited opening. Among them, pain mainly comes from joint trauma, inflammation, joint disc displacement and so on, involving muscle, fascia, bone joint and other tissue structure.^[16]^ When the temporomandibular joint opening movement, it will produce pain or pain aggravation, resulting in limited opening of the patient.^[17]^ According to the disease spectrum study of Tuina, musculoskeletal disease is the dominant disease of Tuina, and Tuina has obvious curative effect on muscle pain, joint disorder and other symptoms.^[18]^ Therefore, for different types of TMD, different Tuina techniques can be used for targeted treatment to improve clinical symptoms. Relevant clinical studies have shown that Tuina can significantly relieve the pain of TMD patients, the number of joint bouncing, and restore the degree of opening of patients.^[19–20]^

Tuina has a good effect on TMD. And Tuina has the characteristics of simple operation and less pain, so it is very suitable for TMD, which is easy to have repeated attacks. At present, there are many clinical studies on Tuina in the treatment of TMD, but its clinical efficacy has not been systematically evaluated, so it is necessary to objectively evaluate the efficacy and safety of Tuina on TMD. Due to the influence of the quality and quantity of the literature included in the study, this study still has its limitations, and because of language ability, search is only included in Chinese and English studies. Therefore, it is necessary to have a large sample and multi-center.

## Author contributions

**Data curation:** Yongchao Li, Lirong Zeng.

**Funding acquisition:** Wenjuan He.

**Funding support**: Wenjuan He.

**Resources:** Wei Sun, Ni Wei.

**Software:** Lirong Zeng, Wei Sun.

**Supervision:** Ni Wei, Hui Xie.

**Writing – original draft:** Jing Chen, Yongchao Li.

**Writing – review & editing:** Jing Chen, Wenjuan He.
